# RACIAL/ETHNIC DIFFERENCES IN NEUROCOGNITIVE PROFILES OF OLDER ADULTS WITH 6-YEAR INCIDENT DEMENTIA

**DOI:** 10.1093/geroni/igad104.3094

**Published:** 2023-12-21

**Authors:** Moyosoreoluwa Jacobs, Carmen Chek, Wassim Tarraf

**Affiliations:** Wayne State University, Detroit, Michigan, United States; Wayne State University, Detroit, Michigan, United States; Wayne State University, Detroit, Michigan, United States

## Abstract

Hispanic and non-Hispanic Black (NHB) older adults have higher dementia rates than non-Hispanic Whites (NHWs). Estimates of dementia prevalence and incidence in community-based cohorts are mostly based on threshold value performance on cognitive assessment batteries. We used longitudinal data from the HRS Harmonized Cognitive Assessment Protocol (HCAP) to (1) generate and compare, across race/ethnicity, neurocognitive profiles for older adults (>65 years) not meeting research criteria for dementia in 2010 and developing dementia by 2016 (using Langa-Weir’s dementia classification), (2) assess racial/ethnic differences in these profiles, and (3) test socio-demographic effects on dementia-specific differences in neurocognitive performance. Performance on all HCAP measures was highly sensitive to 6-year incident dementia classification. However, we found notable domain-specific differences in test sensitivity, whereby performance on general screeners (e.g., MMSE) and memory-based tests (e.g., CERAD word list recognition) were significantly lower (≤2 SDs from normed population means). Hispanics and NHBs generally performed lower than NHWs, with more pronounced group differences in general screeners and executive function tests. Sociodemographic characteristics significantly attenuated Hispanic differences, but less so for NHBs, relative to NHWs. Despite these group differences, NHWs with incident dementia had worse neurocognitive profiles than their NHB and Hispanic counterparts. Dementia classification algorithms (e.g., Langa-Weir) sensitively detect cognitive impairment. However, these algorithms more accurately identify higher severity cognitive impairment in NHWs compared to NHBs and Hispanics. Such methods can yield dementia estimates biased by sociocultural factors. Further research is needed to identify culturally appropriate neurocognitive tests that enhance precise dementia diagnosis, using population-level epidemiological data.

